# Newspaper Notoriety

**Published:** 1898-02

**Authors:** 


### Newspaper Notoriety.

It is very noticeable of late how some physicians in Baltimore have taken pains to let their names and their work appear heralded in the daily papers. In one instance it is in the form of an interview, in another an ordinary operation is commented on as if it were something almost unheard of, and in other ways physicians, with an ill-advised desire to make themselves better known to the public, have allowed their names to appear in the daily press. All such actions savor of bad taste and are not characteristic of the highest state of medicine.

Perhaps the least objectionable form of publicity in the paper is the interview when it is desired to make public the opinion of authorities in medicine on some question which is as yet in doubt. Even this form of notoriety, however, is much abused, and many words are uttered more with the object of attracting the public attention than of answering the questions. The public is more and more interested in anything pertaining to health, and persons everywhere are less inclined to take statements from physicians without asking the reason why. This general thirst for knowledge may be laudable, but a person half instructed is too ready to generalize on too narrow a basis and do more harm than good.

Therefore, for the present at least, it is better to reserve all descriptions for the medical journals, and physicians should use a little discretion in publishing their views and reviewing their work in the daily papers.—*Maryland Medical Journal.*

